# Supplementary figures and images for: The complex role of transcription factor GAGA in germline death during Drosophila spermatogenesis: transcriptomic and bioinformatic analyses
Source: PeerJ. 2023 Jan 9;11:e14063. doi: 10.7717/peerj.14063 (PMC9835689; doi:10.7717/peerj.14063)

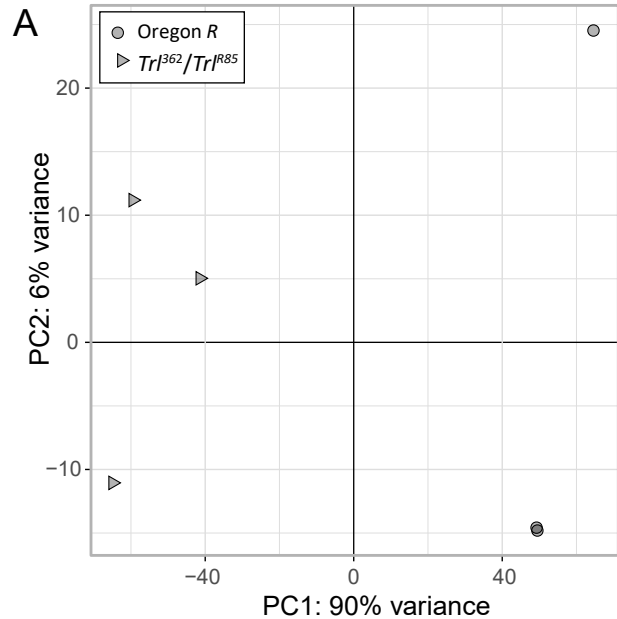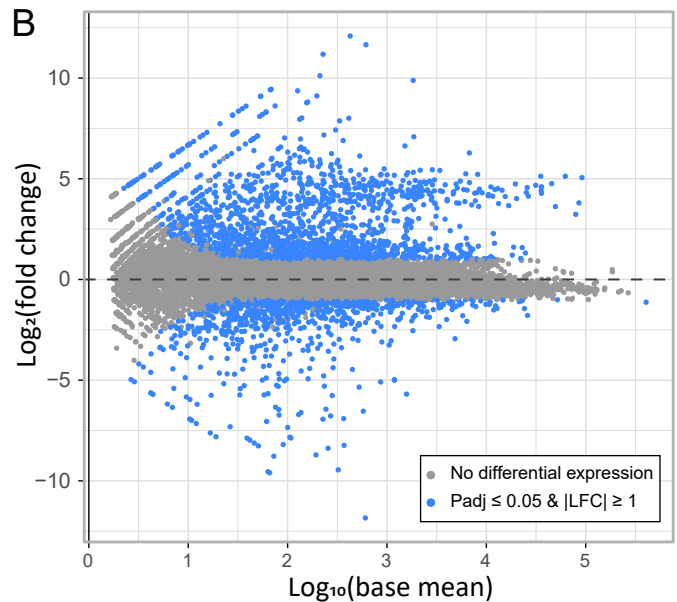

Supplement: Figure S1 — (A) Principal component analysis of the transcriptomic datasets under study. Individual samples are indicated as follows: circles, Oregon R samples; triangles, TrlR85/Trl362 samples. (B) The MA-plot shows log_2FC values between TrlR85/Trl362 and Oregon R samples versus log10-transformed base mean for each gene ID. The x-axis represents the log10-transformed average expression of genes across samples, and the y-axis indicates the log2FC between the TrlR85/Trl362 and Oregon R datasets. Blue circles represent DEGs with statistical significance, p < 0.05 [file peerj-11-14063-s001.pdf]

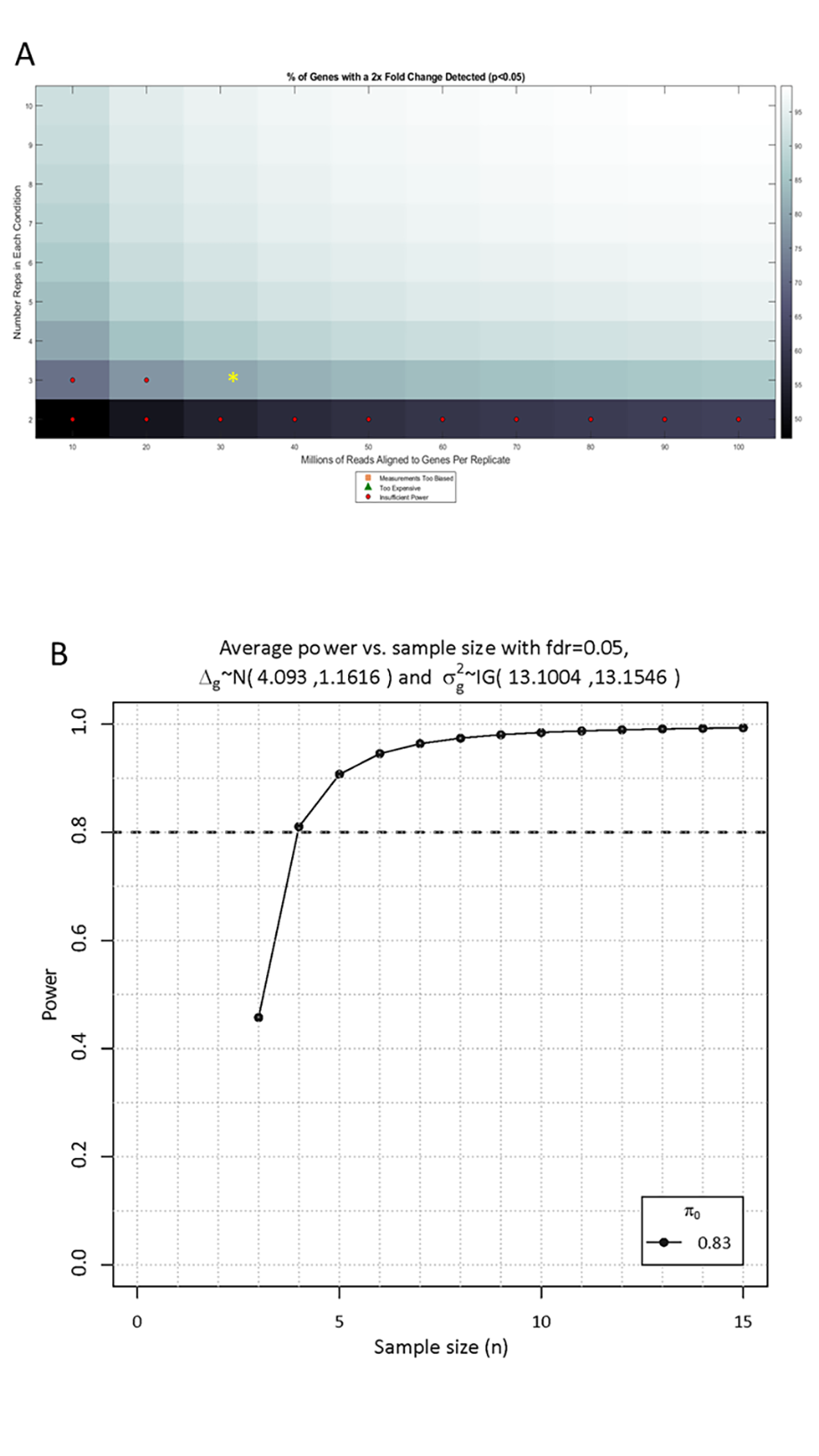

Supplement: Figure S2 — (A) Result from scottyEstimate function (Busby et al., 2013). The current study is indicated by an asterisk. (B) Power calculation result from ssizeRNA 1.3.2 programm (Bi & Liu, 2016). [file peerj-11-14063-s002.png]

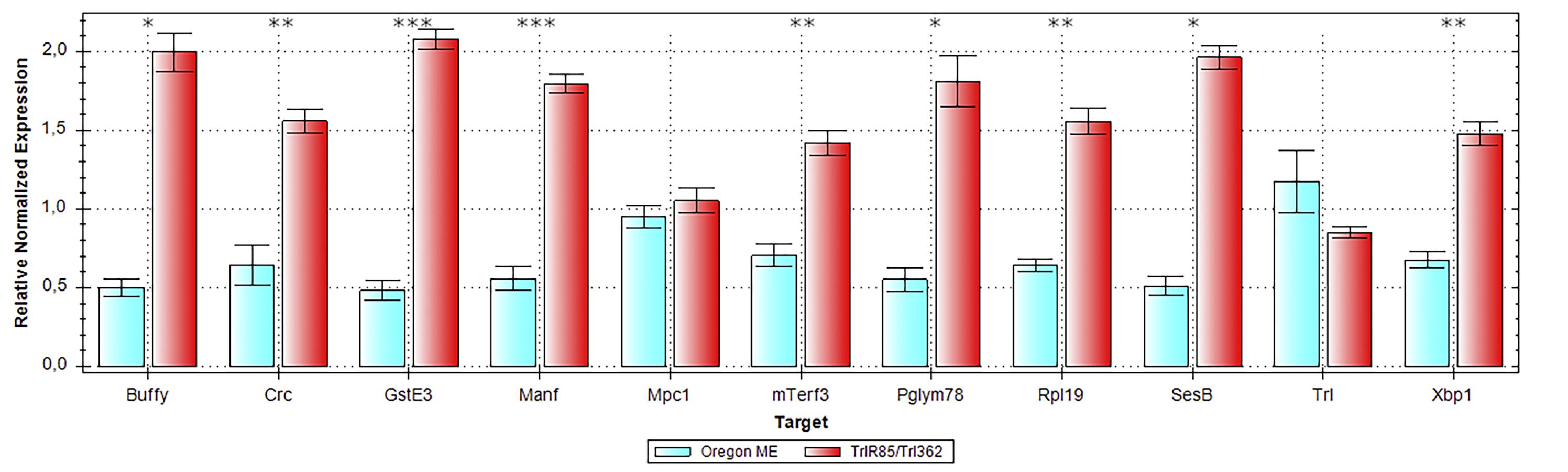

Supplement: Figure S3 — Comparative gene expression data are presented as the mean of gene expression normalized to the expression of the reference genes (in triplicate) ± standard error of the mean. Fzo, Sa, and Mst89b served as reference genes. *—p.adj < 0.05; **—p.adj < 0.01; ***—p.adj < 0.001 [file peerj-11-14063-s003.png]

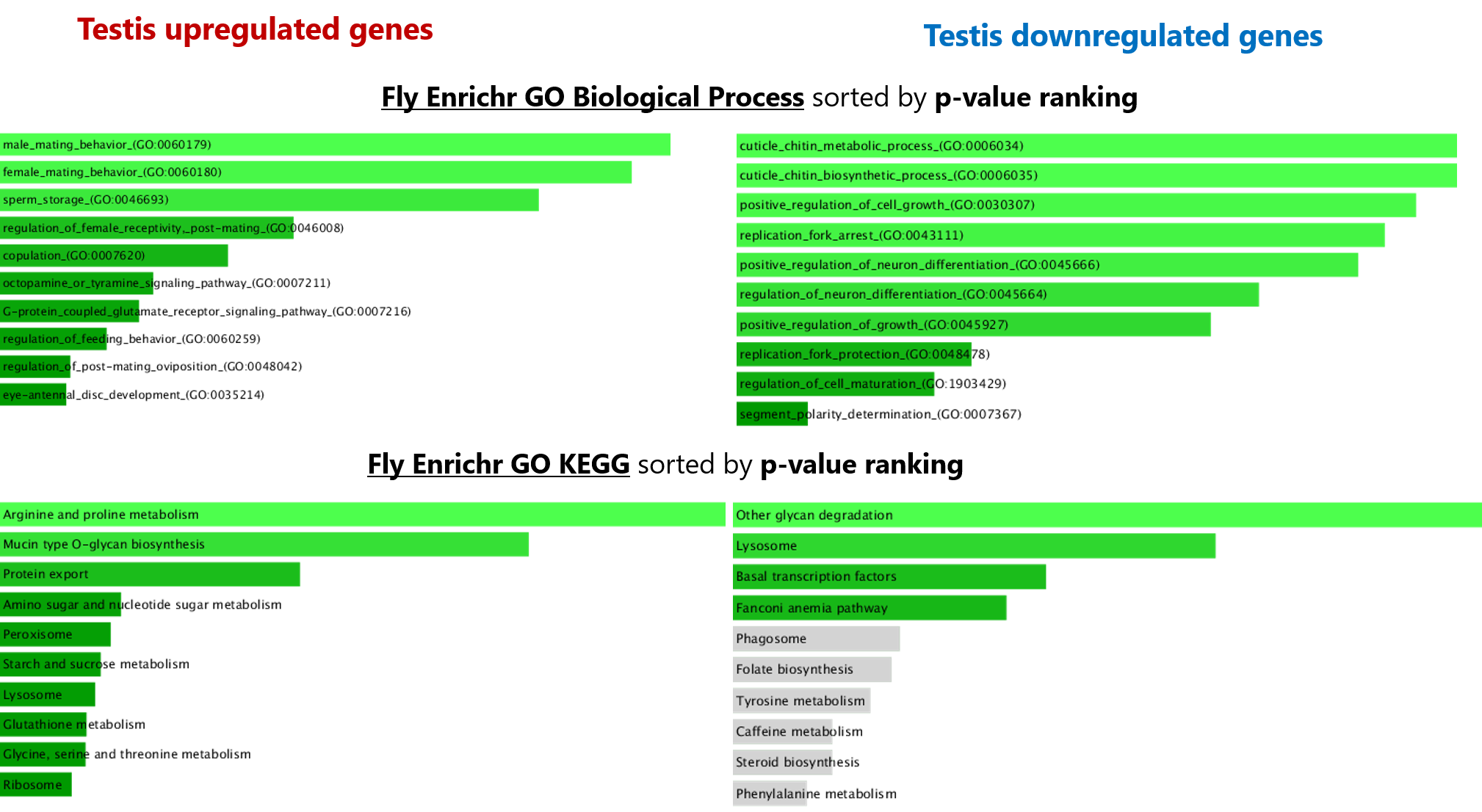

Supplement: Figure S4 — The GO terms most enriched in our sets of upregulated (left column) and downregulated (right column) DEGs [file peerj-11-14063-s004.png]

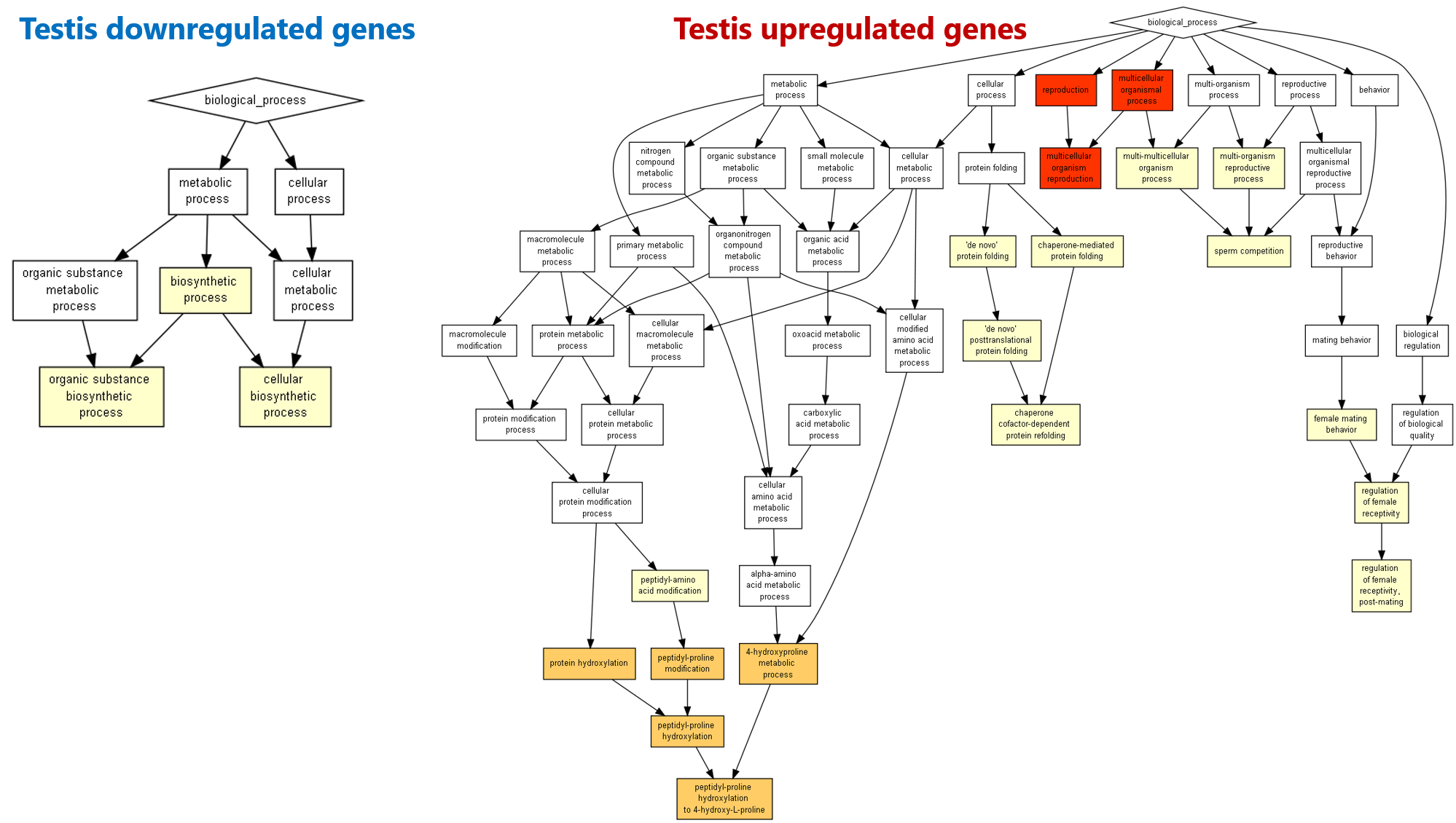

Supplement: Supplemental Information 5 — The sets of up- and downregulated DEGs (Padj ≤ 0.050.05, FC > 2 or FC < 0.5) were tested for GO enrichment on the background of the genes expressed in testes (14,579 genes). The significance threshold (P-value) was set to 10−3 [file peerj-11-14063-s005.png]

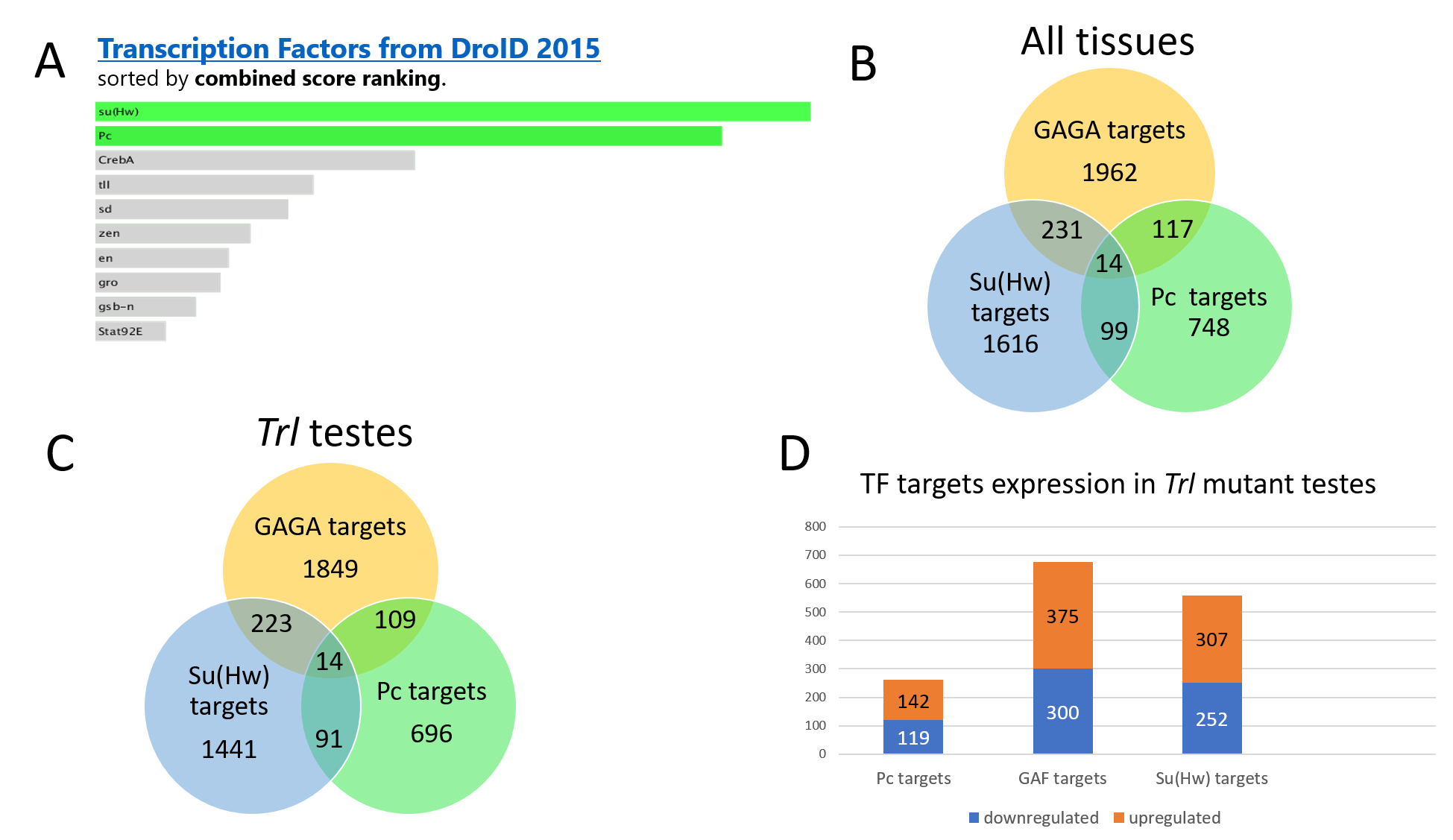

Supplement: Figure S6 — Our set of DEGs upregulated in mutant testes, according to FlyEnrichr analysis (A), is enriched with target genes of transcription factors Pc and Su(Hw). (B) The distribution of target genes of these transcription factors across all Drosophila tissues. (C) The distribution of target genes of Pc, GAGA, and Su(Hw) in fly testes. (D) The proportion of up- and downregulated target genes of the respective factors in Trl-mutant testes. [file peerj-11-14063-s006.png]
