# Supplementary material for: The complex role of transcription factor GAGA in germline death during Drosophila spermatogenesis: transcriptomic and bioinformatic analyses
Source: PeerJ. 2023 Jan 9;11:e14063. doi: 10.7717/peerj.14063 (PMC9835689; doi:10.7717/peerj.14063)
Supplement: Table S1 [file peerj-11-14063-s007.docx]

**Supplementary Table S1. Sequences of primers used for qPCR.**

| Gene symbol | Forward primer | Revers primer | Product length (bp) |
| --- | --- | --- | --- |
| *Rpl19* | 5′-GCGGCAAGAAGAAGGTCTGG-3′ | 5′-ACGACGGGCTTCTTGATGAT-3′ | 117 |
| *Fzo* | 5′-CAAAGAGTAGGTCGGTGCGT-3′ | 5′-CTGGCCTATTTGTGCTGGGA-3′ | 110 |
| *Sa* | 5′-CGATGCTGGAACTTTTGCGT-3′ | 5′-GTGCGCTCCAGATCGAAGTA-3′ | 108 |
| *Mst89B* | 5′-TGCCAAAACGCAGACAACTG-3′ | 5′-AAGTTCTAGTTGGGGTGGCG-3′ | 85 |
| *Crc* | 5′-CGATGCTTACAAACAGGCGG-3′ | 5′-AAGTTAAAGGGCGTGGCAGA-3′ | 119 |
| *SesB* | 5′-TGGTGCTACCTCTCTGTGCT-3′ | 5′-AATTCACGCTGACCACCCTT-3′ | 89 |
| *Xbp1* | 5′-CCTGGGAGGAGAAAGTGCAAA-3′ | 5′-TGTCAGCTCCTTGATCTCGT-3′ | 115 |
| *Mpc1* | 5′-CGCCGACACACAAAAGAGTC-3′ | 5′-GGCTGGACCTTGTAGGCAAA-3′ | 101 |
| *Pglym78* | 5′-AATCAGTTCTGCGGCTGGTA-3′ | 5′-GGGCCACATCGAACTCCAG-3′ | 108 |
| *mTerf3* | 5′-TGCCAAGGAGCTAAGTGACC-3′ | 5′-GGAAGGCCCATATCCTGGTG-3′ | 107 |
| *Manf* | 5′-CTCAACGACTGGGACGAGAG-3′ | 5′-GGGCTTCAGCTCCTCGATAC-3′ | 77 |
| *GstE3* | 5′-AAGACGGAGATTCCACAGGC-3′ | 5′-ATCGGCAATGGTCAGGTTGT-3′ | 113 |
| *Trl* | 5′-ATCCACGCCAAAGGCAAAAC-3′ | 5′-GCAAATGGGGCAAGTAGCAG-3′ | 96 |
| *Buffy* | 5′-ATGTGTTTCCAGCGGTCCAA-3′ | 5′-TATGGAACTCACCACCCGGA-3′ | 110 |
