# Supplementary material for: The complex role of transcription factor GAGA in germline death during Drosophila spermatogenesis: transcriptomic and bioinformatic analyses
Source: PeerJ. 2023 Jan 9;11:e14063. doi: 10.7717/peerj.14063 (PMC9835689; doi:10.7717/peerj.14063)
Supplement: Table S2 — FC was assessed as the ratio of expression in mutant vs. wild-type flies. The p values were calculated by Student’s t test. Correction for multiple comparisons was performed by the Benjamini–Hochberg method [file peerj-11-14063-s008.docx]

**Supplementary Table S2. qPCR results.**

FC was assessed as the ratio of expression in mutant vs. wild-type flies. The p values were calculated by Student’s *t* test. Correction for multiple comparisons was performed by the Benjamini–Hochberg method.

| **genes** | **Fold_change_qPCR** | **qPCR P_value_** | **qPCR P_adj_** | **Fold_change RNA-seq** | **RNA-seq P_value_** | **RNA-seq P_adj_** |
| --- | --- | --- | --- | --- | --- | --- |
| *RpL19* | 2.44 | 8.22E-04 | 5.63E-03 | 1.71 | 4.15E-05 | 2.43E-04 |
| *Buffy* | 3.98 | 6.67E-03 | 2.67E-02 | 2.78 | 1.46E-09 | 1.91E-08 |
| *crc* | 2.43 | 7.66E-04 | 5.63E-03 | 1.53 | 6.29E-04 | 2.72E-03 |
| *GstE3* | 4.32 | 4.74E-06 | 5.21E-05 | 2.11 | 2.15E-05 | 1.34E-04 |
| *Manf* | 3.22 | 9.98E-05 | 9.98E-04 | 2.33 | 1.20E-07 | 1.16E-06 |
| *mTerf3* | 2.01 | 7.04E-04 | 5.63E-03 | 2.48 | 1.61E-06 | 1.28E-05 |
| *Mpc1* | 1.11 | 3.16E-01 | 3.16E-01 | 1.14 | 2.41E-01 | 3.80E-01 |
| *Pglym78* | 3.28 | 1.25E-02 | 3.74E-02 | 2.03 | 4.55E-08 | 4.74E-07 |
| *sesB* | 3.86 | 2.24E-03 | 1.12E-02 | 2.04 | 8.76E-09 | 1.03E-07 |
| *Trl* | 0.72 | 1.29E-01 | 2.58E-01 | 1.63 | 3.57E-02 | 8.46E-02 |
| *Xbp1* | 2.18 | 2.81E-04 | 2.53E-03 | 1.52 | 6.83E-04 | 2.91E-03 |
